# Stenting the Superior Petrosal Sinus in a Patient With Symptomatic Superior Semicircular Canal Dehiscence

**DOI:** 10.3389/fneur.2018.00689

**Published:** 2018-08-20

**Authors:** Eugen C. Ionescu, Aurelie Coudert, Pierre Reynard, Eric Truy, Hung Thai-Van, Aicha Ltaief-Boudrigua, Francis Turjman

**Affiliations:** ^1^Service Audiologie et Explorations Otoneurologiques, Hospices Civils de Lyon, Lyon, France; ^2^Service ORL, de Chirurgie Cervico-Faciale et d'Audiophonologie, Hôpital Femme Mère Enfant, Hospices Civils de Lyon, Lyon, France; ^3^Service ORL et Chirurgie Cervico-Faciale et d'Audiophonologie, Hôpital Edouard Herriot, Hospices Civils de Lyon, Lyon, France; ^4^IMPACT Team, INSERM U1028 Centre de Recherche en Neurosciences de Lyon, Lyon, France; ^5^Service d'Imagerie Médicale et Interventionnelle, Hôpital Edouard Herriot, Groupement Hospitalier Est, Hospices Civils de Lyon, Lyon, France; ^6^Service d'Imagerie Médicale Neuro-Interventionelle, Hôpital Neurologique, Hospices Civils de Lyon, Lyon, France; ^7^UMR5515, INSERM U1206 Centre de Recherche en Acquisition et Traitement d'Images pour la Santé (CREATIS), Villeurbanne, France

**Keywords:** third window lesions, semicircular canal dehiscence, superior petrosal sinus, endovascular treatment, pulsatile tinnitus

## Abstract

Patients presenting superior semicircular canal dehiscence (SSCD) can experience symptoms such as conductive hearing loss, pulsatile tinnitus, autophony, and pressure-induced vertigo. Decreased cervical vestibular-evoked myogenic potentials (cVEMPs) thresholds and high-resolution computed tomography (HRCT) of the petrous bone are essential for diagnosis of SSCD syndrome. We report the case of a 43-year-old man suffering from constant right pulsatile tinnitus, intermittent autophony, and unsteadiness induced by physical exercise. An SSCD by the superior petrosal sinus (SPS) was confirmed on the right side by axial HRCT of the temporal bone reformatted in the plane of Pöschl and ipsilateral abnormally low elicited cVEMPs. Treatment options were discussed with the patient since the pulsatile tinnitus progressively became debilitating. Two options were considered: surgery or a new endovascular treatment; the patient chose the latter option. After stenting the right SPS, the intensity of the pulsatile tinnitus dramatically decreased. As there was no complication the patient was discharged at Day 1. The other symptoms improved progressively. By the 60-day follow-up visit the patient only reported a slight tinnitus worsened by physical exercise. Angiographic follow-up at 5 months confirmed the patency of the SPS. Stenting the SPS in patients with SSCD by the SPS appears to be an alternative to the existing surgical treatments.

## Background

The superior semicircular canal dehiscence (SSCD) syndrome was described by Minor in 1998 (1). In a survey study that included 1,000 temporal bones a SSCD was identified in five specimens from four adults (0.5%) (2). Among these, one SSCD was located between the superior semicircular canal (SSC) and the middle cranial fossa and four were found in relation with the superior petrosal sinus (SPS); only one such subject complained about audio-vestibular symptoms during life. In a review of the literature (3) SSCD by the SPS accounted for 4–9% of ears in symptomatic SSCD. This variant of SSCD may be associated with higher rates of pulsatile tinnitus (4).

The main symptoms of SSCD by the SPS are: conductive hearing loss, pulsatile tinnitus, autophony, and pressure-induced vertigo (3–8). As previously reported, decreased cervical vestibular-evoked myogenic potentials (cVEMPs) thresholds are considered as a specific sign for third window lesions (9) and, HRCT is essential to identify the characteristic bony groove of the SPS out of the SSCD (3).

To date, surgery is the only effective treatment in patients with disabling SSCD by the SPS. Surgical techniques include plugging, resurfacing, and capping of the SSC, and these are frequently combined for a better result. Middle cranial fossa and transmastoid approaches are the two main surgical options (10–12). Resurfacing and/or plugging via a middle fossa craniotomy in patients with SSCD by SPS implies mobilizing the SPS which may cause bleeding or complications related to the surgical approach (6, 7, 11). Although this maneuver can be difficult, the method has been reported to lead to good post-operative results in terms of vestibular symptoms (6, 10, 11, 13). The transmastoid approach is preferred by some authors in case of SSCD by the SPS (10, 12) since it is safer with lower risks for the integrity of the SPS.

Plugging the affected superior semicircular canal appears to be the most effective technique for improving symptoms; however, the main criticism of plugging is that it requires opening or manipulating the labyrinth, with variable impact on ipsilateral labyrinthine function. We therefore propose an alternative endovascular technique designed to reduce the dissipation of acoustic energy through the third window and to preserve the vestibular function. To the best of our knowledge, the case presented herein is the first SSCD patient to have benefited from an endovascular treatment.

## Case history and procedure

A 43-year-old male was referred to our otolaryngology department for constant right pulsatile tinnitus, intermittent autophony, and unsteadiness induced by physical exercise. The patient did not have any medical history before the progressive onset of the symptoms. He underwent successive clinical examinations, as well as auditory and vestibular assessment as follows: Pure Tone Audiometry (PTA; Madsen Astera-Otometrics) middle ear reflexes (Madsen Zodiac 901 tympanometer), videonystagmography (VNG, Ulmer System®; Synapsis SA), video head impulse test (VHIT, ICS Impulse®; GN Otometrics), and cVEMPs (Bio-Logic® Nav-Pro system) in air conduction stimuli with 750 Hz tone bursts. The patient completed before intervention a Tinnitus Handicap Inventory (THI) questionnaire adapted to French language. Another THI questionnaire was completed after the endovascular procedure at Day 1 and 60.

A HRCT (GE GSI Revolution; GE Healthcare) of the petrous bone was performed. Slices were acquired helically in the axial plane, at a nominal 0.625 mm slice thickness with a 50% overlap of 0.312 mm as recommended when a SSCD syndrome is suspected (14). All images were obtained in ultrahigh resolution at 140 kV and 200 mAs/section. The primary images were retargeted to the axial and coronal planes of the lateral semicircular canal, to a 60 mm field of view with a 512 matrix for an isometric voxel. The retargeted axial scans were then reformatted in the Pöschl plane, using AW Server software, GE Healthcare.

## Results

**Figure 1:**
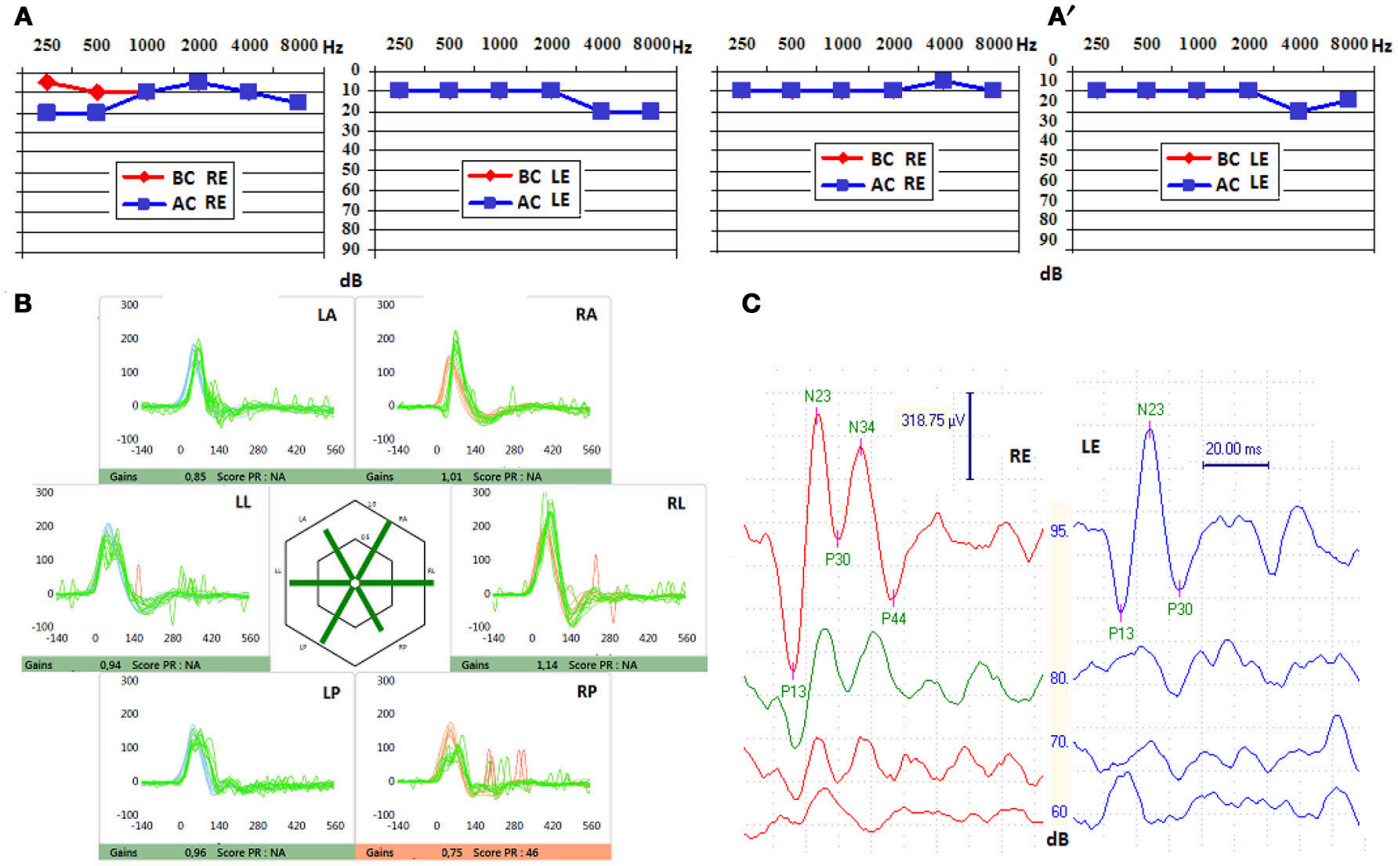
**(A)** Pure tone audiometry before stenting, showing a right air-bone gap affecting low frequencies, that disappeared after endovascular approach **(A**′**)**. BC, bone conduction; AC, air conduction; RE, right ear; LE, left ear. **(B)** Video Head Impulse Test (VHIT) after stenting, with unchanged gains for all semi-circular canals. LA, left anterior; LL, left lateral; LP, left posterior; RA, right anterior; RL, right lateral; RP, right posterior. **(C)** Cervical vestibular evoked myogenic potentials (cVEMPs) found before stenting, unchanged after fitting the stent in the superior petrosal sinus (SPS), showing lowered threshold (valued at 70 dB SPL) on the right side (red). RE, right ear; LS, left ear.

**Figure 2:**
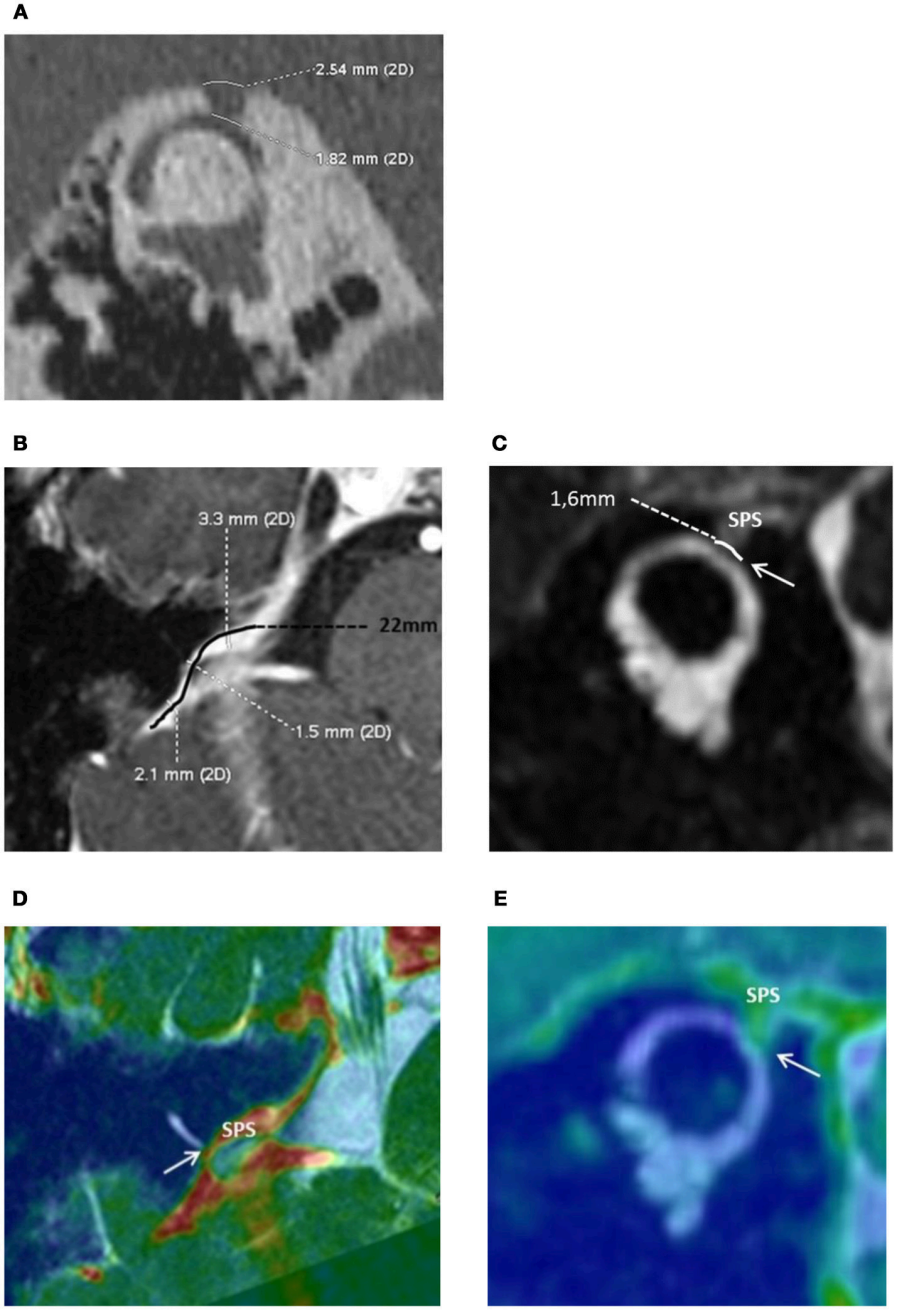
**(A)** High resolution computed tomography of the right petrous bone. Characteristic “Cookie bite” sign (3) in the Pöschl plane indicating superior semicircular canal (SSC) dehiscence by the Superior Petrosal Sinus (SPS). The bone defect at the dehiscent level between the SSC and the SPS was 1.82 mm in length. **(B)** Magnetic Resonance Imagery of the right inner ear. The Superior petrosal sinus (SPS) and the Superior Semicircular Canal (SSC): anatomical reports and measurements. Axial 3D T1 weighted enhanced contrast. The length of the right SPS was 22 mm—“S” shape curved black line. Minimal SPS internal diameter in contact with the SSC was measured at 1.5 mm. **(C)** Coronal oblique T2 high resolution FIESTA, Pöschl plane incidence. SPS wall in contact with the membranous SSC through a limited zone of about 1.6 mm (white arrow). Merged, 3D T1 weighted enhanced contrast and FIESTA axial **(D)** and coronal oblique sequences **(E)**.

The audio-vestibular assessment was normal except a right-sided low frequency conductive hearing loss (15 dB HL at 250 Hz and 10 dB HL at 500 Hz; Figures 1A,B), and an ipsi-lateral abnormally low cVEMPs threshold at 70 dB SPL (Figure 1C). THI questionnaire score before treatment was of 80/100 indicating severe handicap according to the classification reported by Newman et al. (15). HRCT confirmed a bony defect on the right SSC limited to the groove of the SPS (Figure 2A).

A brief clinical improvement occurred several months after a T-tube insertion in the right tympanic membrane; however, the patient continued to complain of disabling pulsatile tinnitus that greatly impaired quality of life. The frequency of autophony symptoms progressed and he began to experience a sensation of “moving eyeballs” when speaking loudly. As a result of his symptoms, the patient could no longer work, and became depressed. Surgical options and risks were discussed with the patient. Since surgery was thought too invasive by the patient an alternative endovascular treatment was considered by the medical staff.

## Further exams and intervention

**Figure 3:**
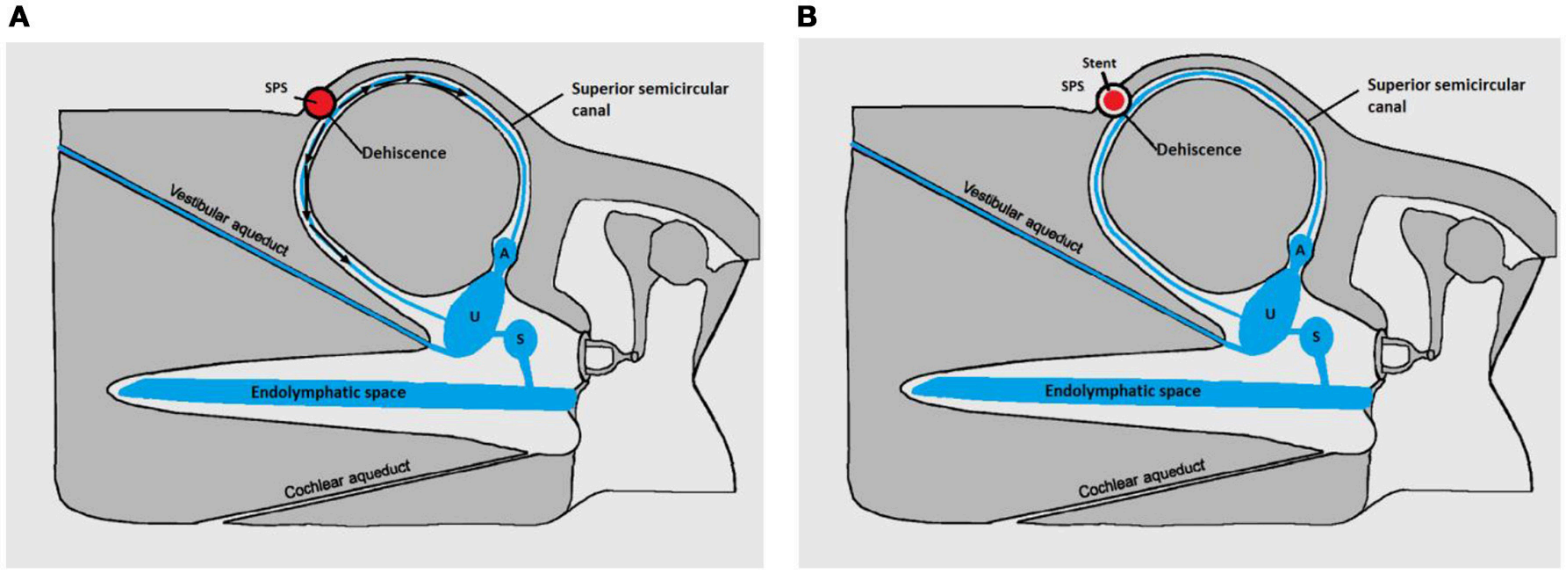
Pathomecanism of SSCD by the SPS-modified with permission from Merchant and Rosowski (8). **(A)** Before stenting. Endolymphatic flow (black arrows) generated by the wall pulsations of the superior petrosal sinus (SPS) in contact with the membranous SSC resulting in abnormal auditory and vestibular stimulation. **(B)** After stenting. Principle of the endovascular treatment: rigidifying the SPS wall would diminish the energetic transfer between the vascular structure and the perilymphatic/endolympatic compartments. Cochlear and vestibular ends organs are no longer abnormally stimulated. U, utricle; S, Saccule; A, Ampulla of the SSC.

The patient underwent a cerebral Magnetic Resonance Angiography (MRA, 3T MRI–MR750 Discovery, GE Healthcare) and a cerebral angiogram (Monoplane angiographic system, ALLURA 400^©^, Philips). As the cerebral and peri-petrosal vascular system was normal, the feasibility of an endovascular procedure was considered. The two main challenges were: (1) access and (2) rigidify the SPS wall in contact with the labyrinthine membrane (Figures 2A–E). The objective was to attenuate the pulsations of the SPS wall transmitted through the SSCD to the perilymphatic/endolymphatic compartments which then would abnormally stimulate cochlea and vestibular end organs (8) (Figure 3A). As a more rigid SPS wall would increase the impedance at the dehiscence level, it was planned to fit a stent of convenient dimensions in the middle third of the venous structure in contact with the SSC (Figures 2C–E, 3B). The entire procedure was explained to the patient who agreed with this new treatment.

**Figure 4:**
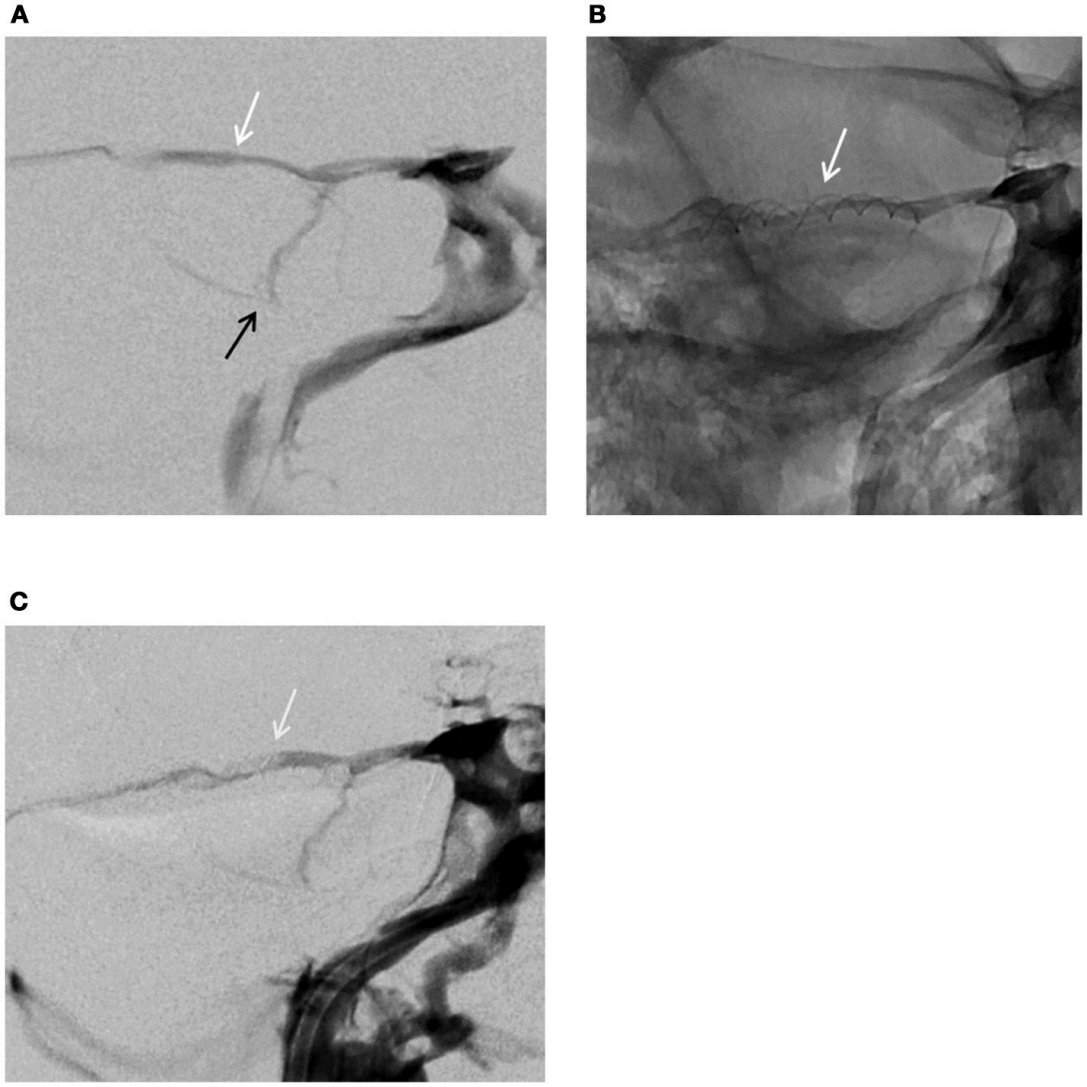
Venogram–frontal views. **(A)** Normal venous configuration before stenting the SPS: white arrow (SPS), black arrow (superior petrosal vein) **(B)** Stents fitted in the SPS (white arrow) **(C)** Venogram at 5 months showing the patency of the superior petrosal sinus, stents fitted in the SPS (white arrow).

A standard femoral vein puncture was made under general anesthesia and 500 mg of IV aspirin was injected per procedure. A six French guiding catheter with guide wire was moved forward into the right inferior petrosal sinus, guided by digital subtraction imaging. After accessing the right cavernous sinus, a venogram with retrograde opacification confirmed the presence of a complete petrosal venous system (Figure 4A). A 0.010-inch microcatheter with micro guide wire (Echelon; Medtronic) was inserted inside the SPS and a selective venogram was obtained. The minimal diameter of the SPS in the planned stenting zone was measured at 1.5 mm. It was decided to place a 2 × 20 mm Leo stent (Balt, Montmorency, France). The placement of the stent was uneventful but considered too distal. A second stent (2 × 12 mm) was placed more proximally at 1 cm distal to the cavernous sinus avoiding obstructing the Superior Petrosal Vein (SPV) junction to the SPS (Figure 4B). At the end of the procedure, a venogram demonstrated the patency of both SPS and SPV. Guiding and microcatheter were withdrawn and manual pressure was applied at the groin.

When the patient awoke up from general anesthesia autophony and pulsatile tinnitus had dramatically decreased. The THI questionnaire completed at Day 1 dropped to 54 points indicating a moderate handicap. Exercise-induced vertigo and dizziness progressively disappeared, and the patient was able to return to work a few weeks thereafter. At month 1 the initial air-bone gap (ABG) on the right was normalized although right cVEMPs threshold remained unchanged. VNG was normal with no evidence of canal paresis. VHIT results were not different of those found before treatment (Figure 1B). The THI questionnaire score at Day 60 was 20 points indicating a mild handicap. Angiographic follow-up at 5 months confirmed the patency of the petrosal venous system (Figure 4C). The patient had to follow a preventive antithrombotic treatment (75 mg aspirin and 75 mg clopidogrel) for 3 months, then 75 mg daily aspirin daily for an additional 6 months. This is the standard protocol proposed by our neuro-interventional team after treating cerebral aneurysms or arterio-venous malformations to prevent secondary thrombosis. The audio-vestibular findings remain stable at month 5.

## Discussion

A 43-year-old man presented a typical SSCD syndrome related to SPS. The main auditory complaints were: constant pulsatile tinnitus and intermittent autophony. He also mentioned exercise-induced unsteadiness and showed progressively signs of depression that have been frequently reported in case of persistent tinnitus (16, 17). Since T-tube insertion failed to satisfactorily alleviate the symptoms and the patient reported that the symptoms were debilitating, two possible interventions were discussed with the patient.

The first was the surgical option. Surgical techniques such as resurfacing, plugging (or both combined) of the SSCD by a middle-fossa approach have been frequently reported in the literature (6, 7, 10, 11, 13). However, only a few patients have been surgically treated for SSCD by the SPS (3, 6, 7, 11, 13). Cheng et al. (18) emphasized the difficulties and potential complications when using bone wax for plugging the SSC. Despite the sealing of the dehiscence, if a too large quantity of wax extends along the canal anteriorly toward to the ampulla or posteriorly to the common crus, patients will experience vestibular and hearing impairments after surgery. Indeed, post-surgical vestibular impairments of various degrees are frequent since the principle of plugging is to surgically occlude the SSC (10, 12, 13, 15, 16, 19, 20). This inconvenience also applies in cases of plugging by the transmastoid approach since the procedure is the same. The second was the endovascular option. This technique could rigidify the SPS wall in contact with the labyrinth by fitting a stent slightly wider than the minimal diameter of the vein (Figure 3). As the vascular anatomy of the region was normal, the risks per procedure related to this technique were acceptable for the patient.

Bleeding or thrombosis of the SPS were two possible local complications in this technique. The risk of bleeding was estimated as minimal since the petrosal venous system was complete allowing a relatively easy retrograde access of the guiding catheter in the SPS via the cavernous sinus. This approach is frequently used by our neuro-interventional team in case of other neuro-vascular pathologies. The deployment of both stents in the lumen of the SPS was gentle and uneventful due to the patency of the SPS at the end of the procedure. The risk of an eventual secondary thrombosis on the stent was estimated low since the venous disposition through the cavernous sinus and to the inferior petrosal sinus was normal in shape and dimensions. In case of any local venous perturbation generated by the presence of the stent in the middle third of the SPS, an alternative drainage of the SPV through the jugular vein would still be possible in a retrograde way (via the cavernous sinus and/or the inferior petrosal sinus) with no hemodynamic impact. However, in case of important anatomical variations of the venous petrosal system (e.g., narrowed or/and hypoplastic inferior or SPS) stenting the SPS would not be recommended. The reasons that finally accounted for the decision to perform the endovascular treatment were: a less invasive and (we believe) safer procedure for the inner ear, and the presence in our team of an experienced interventional-radiologist with particular expertise in neuro-vascular pathologic conditions affecting the peri-petrosal venous system. The patient recovered rapidly and was discharged the day after the procedure. The early Day 1 THI questionnaire showed a notable improvement that further improved by Day 60. Right cVEMPs threshold remained abnormally low although ipsi-lateral ABG normalized (Figure 1A′), and the vestibular assessment remained normal as prior to the procedure. A possible explanation for these findings would be that the procedure was designed to reduce the transmission of the pulsatile energy through the perilymphatic/endolymphatic compartments by rigidifying the SPS wall. As a consequence, the impedance added by the stent would be sufficient to suppress the slight right audiometric conductive gap of 15–25 dB observed initially. However, as the third window is not completely excluded as in case of surgery, the right cVEMPs threshold remains low since they are elicited at much higher acoustic pressure levels.

## Conclusion

In this patient with symptomatic SSCD by the SPS, the endovascular treatment appears to be an effective procedure. Although stenting the SPS was successful in this case, in the future similar cases should be carefully selected before proposing this approach. The main advantages which seem to emerge using this technique are the preservation of the vestibular function, a limited risk for hearing, and a limited risk of bleeding since the elastic tension exerted by the stent on SPS wall would be minimal and uniformly applied on the internal surface of the vessel. As for the surgical treatment, this technique should be reserved for very impaired patients. Normal anatomy of the petrosal and jugular venous system is necessary and should be determined by selective venogram prior to the procedure. Antithrombotic therapy for 9 months after the procedure would add minimal risk, but is important in order to avoid secondary thrombosis of the stent.

## Ethics statement

This study/case report was carried out in accordance with the recommendations of Haute Autorité de Santé (https://www.has-sante.fr/portail/jcms/c_2036961/en/best-practice-guidelines), France. A written informed consent was obtained from the patient.

## Author contributions

EI imagined and elaborated the principle of the endovascular treatment. AC elaborated the diagnosis and wrote the manuscript with EI. Her contribution to this paper should be considered similar to EI. PR participated to the elaboration of the treatment principle and adapted with permission the scheme of the third window mechanism. ET, the surgeon of the team, he observed the patient evolution before and after the insertion of the ventilation tube in the right tympanic membrane. HT-V performed and verified the pertinence of the audio-vestibular assessment before and after the endovascular treatment. AL-B, the radiologist of the team referent for the pathology of the middle and inner ear pathology; she performed all specific radiologic exams and prepared the figures of this case report. FT with EI imagined the best approach to the right superior petrosal sinus and realized the procedure; his expertise as interventional neuroradiologist was decisive for the final decision to stent the superior petrosal sinus.

### Conflict of interest statement

The authors declare that the research was conducted in the absence of any commercial or financial relationships that could be construed as a potential conflict of interest.
